# Engineering of B800 bacteriochlorophyll binding site specificity in the *Rhodobacter sphaeroides* LH2 antenna

**DOI:** 10.1016/j.bbabio.2018.11.008

**Published:** 2019-03-01

**Authors:** David J.K. Swainsbury, Kaitlyn M. Faries, Dariusz M. Niedzwiedzki, Elizabeth C. Martin, Adam J. Flinders, Daniel P. Canniffe, Gaozhong Shen, Donald A. Bryant, Christine Kirmaier, Dewey Holten, C. Neil Hunter

**Affiliations:** a Department of Molecular Biology and Biotechnology, University of Sheffield, Sheffield S10 2TN, UK; b Department of Chemistry, Washington University, St. Louis, MO 63130-4889, USA; c Department of Biochemistry and Molecular Biology, The Pennsylvania State University, University Park, PA 16802, USA

**Keywords:** *Rhodobacter sphaeroides*, LH2, Bacteriochlorophyll, Chlorophyll, Light harvesting, Ligand binding, Protein engineering

## Abstract

The light-harvesting 2 complex (LH2) of the purple phototrophic bacterium *Rhodobacter sphaeroides* is a highly efficient, light-harvesting antenna that allows growth under a wide-range of light intensities. In order to expand the spectral range of this antenna complex, we first used a series of competition assays to measure the capacity of the non-native pigments 3-acetyl chlorophyll (Chl) *a*, Chl *d*, Chl *f* or bacteriochlorophyll (BChl) *b* to replace native BChl *a* in the B800 binding site of LH2. We then adjusted the B800 site and systematically assessed the binding of non-native pigments. We find that Arg_−10_ of the LH2 β polypeptide plays a crucial role in binding specificity, by providing a hydrogen-bond to the 3-acetyl group of native and non-native pigments. Reconstituted LH2 complexes harbouring the series of (B)Chls were examined by transient absorption and steady-state fluorescence spectroscopies. Although slowed 10-fold to ~6 ps, energy transfer from Chl *a* to B850 BChl *a* remained highly efficient. We measured faster energy-transfer time constants for Chl *d* (3.5 ps) and Chl *f* (2.7 ps), which have red-shifted absorption maxima compared to Chl *a*. BChl *b*, red-shifted from the native BChl *a*, gave extremely rapid (≤0.1 ps) transfer. These results show that modified LH2 complexes, combined with engineered (B)Chl biosynthesis pathways *in vivo*, have potential for retaining high efficiency whilst acquiring increased spectral range.

## Highlights

• The *Rba*. *sphaeroides* LH2 B800 site preferentially binds BChl *a* over other (B)Chls.
• The BChl *a* 3-acetyl H-bond and B-ring structure contribute to B800 site specificity.
• Removal of H-bonding by βArg_−10_ abolishes specificity for BChl *a* over Chl *a*.
• Non-native (B)Chls in the B800 site rapidly and efficiently transfer energy to B850.
• Engineering LH2 is a potential route to in vivo utilisation of non-native (B)Chls.

## 1 Introduction

The phototrophic purple bacterium *Rhodobacter sphaeroides* (Rba. sphaeroides) utilises light to generate ATP within specialised membrane compartments [1,2] that contain four major complexes, light-harvesting complex 2 (LH2), reaction-centre light-harvesting 1-PufX complex (RC-LH1-PufX), cytochrome *bc*_1_ and ATP synthase [3,4]. Light is absorbed by either the LH2 or LH1 antenna, arranged in protein-rich arrays, and excitation energy is transferred within picoseconds to the RC, where charge separation drives the reduction of quinone to quinol and the oxidation of cytochrome *c*_2_ [[5], [6], [7], [8], [9], [10]]_._ These products are utilised by the cytochrome *bc*_1_ complex, which resides in a locally lipid-rich region, to re-reduce oxidised cytochrome *c*_2_ and oxidise quinol to quinone [3,4,11,12]. This cyclic process results in the transport of protons into the lumen (periplasm) of the chromatophore, which drive ATP generation by ATP synthase [3,4].

**Fig. 1:**
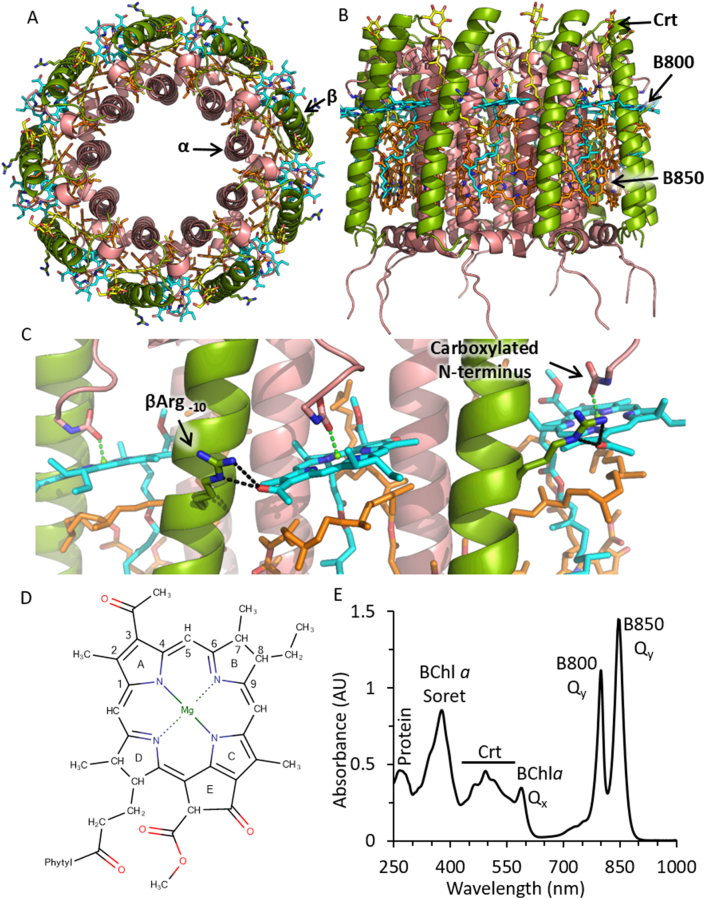
Crystal structure of the *Rbs*. *acidophilus* LH2 complex (PDB ID:1NKZ) [14]. The α and β polypeptides are drawn as cartoons in pink and green, respectively. The βArg_−10_ residue, the carboxylated N-terminus of the α-subunit, and bound cofactors are drawn with sticks. B800 BChl *a* is cyan, B850 BChl *a* is orange and the carotenoid (Crt), rhodopin glucoside, is yellow. Annotated views from the top (panel A), side (panel B) and of the B800 site (Panel C) are shown. H-bonds to the βArg_−10_ residue are shown with dashed black lines, and ligation of Mg by the carboxylated N-terminus is shown with green dashed lines. Panel D shows a 2D schematic of the BChl *a* structure annotated with ring lettering and numbering of porphyrin carbons 1–9. Panel E shows an annotated UV/Vis/NIR absorption spectrum of *Rba*. *sphaeroides* LH2.

LH2 is comprised of nine pairs of α and β polypeptides arranged in a ring. Each αβ pair binds three bacteriochlorophyll *a* (BChl a) molecules, two of which form a strongly excitonically coupled dimer, in which their tetrapyrrole rings are aligned perpendicular to the membrane. A ring of nine of these dimers produces the absorption band at 850 nm, so these BChls are termed B850. The third BChl *a* is bound with its tetrapyrrole ring nearly parallel to the membrane; the ring of these monomeric and weakly coupled BChls gives rise to the absorption band at 800 nm, so these pigments are designated as B800. One carotenoid (Crt), which interacts with both B800 and B850 is also bound to each B800-B850 unit [[13], [14], [15]]. Fig. 1A and B show the structure of the *Rhodoblastus acidophilus* (Rbs. acidophilus) (formerly *Rhodopseudomonas acidophila*) LH2 complex [14]. Fig. 1C shows a detailed view of the B800 site. The B800 BChl *a* (see Fig. 1D for an annotated structure) has several key interactions with the protein. The carboxylated N-terminus of the α-subunit chelates the BChl *a* central magnesium. In *Rba*. *sphaeroides* this post-translational modification is not found, and ligation is likely to be provided by the side-chain of Asn-3 [16]. Site-directed mutagenesis showed that βHis21 is also important, and alteration to Ser abolished B800 binding [17]. Another interaction of note is a hydrogen-bond (H-bond) between βArg_−10_ (βArg30 in *Rba*. *sphaeroides*, which occurs 10 residues before the B850 chelating histidine; this residue is conserved in all LH2 complexes and can be used as a reference point by numbering as His_0_). Mutagenesis of *Rba*. *sphaeroides* LH2 shows that the βArg_−10_ H-bond is dispensable for BChl *a* binding but is required for the majority of the red-shift of its absorption to 800 nm (from 770 nm when BChl *a* is free in solution) and its increase in intensity. These characteristics are mainly due to rotation of the dihedral angle of the 3-acetyl group, which brings it in-plane with the porphyrin macrocycle extending the delocalisation of its electrons, which leads to a bathochromic shift and narrowing of the absorption band [[17], [18], [19], [20], [21]].

Fig. 1E shows the UV/Vis/NIR absorption spectrum of *Rba*. *sphaeroides* LH2. There is strong absorption at 365 nm from the BChl *a* Soret band, broad absorption between 425 and 550 nm from the spheroidene/spheroidenone carotenoids, and a small peak at 595 nm from the BChl *a* Q_x_ transition. In the near infrared (NIR) are two strong peaks from the Q_y_ transitions of the B800 and B850 BChl *a*. The red region of the spectrum between 600 and 750 nm contains no absorption bands, a property shared with RC-LH1-PufX [22]. This so-called red-gap is an adaptation to the ecological niche of these bacteria as they reside deep in water columns below red-light absorbing plants, algae and cyanobacteria that primarily use Chl *a* for phototrophy. Thus, shifting of the main absorption bands to the NIR avoids competition and avoids absorption by water.

Several properties of *Rba*. *sphaeroides* make it a compelling organism for biotechnological applications. It naturally produces dihydrogen [23] and has been genetically engineered to produce a variety of useful compounds, such as alternative carotenoids [24]. For man-made applications where homogeneous cultures are grown, the competition for light is removed and wavelengths within the red-gap are wasted. Attempts to augment the absorption of *Rba*. *sphaeroides* have been made by attachment of yellow fluorescent protein to the RC in RC-LH1-PufX [25], or by red-shifting BChl *a* absorption via addition of a calcium-ion binding site from a related purple bacterial species [[26], [27], [28]]. The pigments housed within the antenna complexes have been altered by engineering carotenoid biosynthesis to produce non-native carotenoids [24]. Chemical modification of LH2 [29,30], LH1 [[30], [31], [32], [33]] and RCs [34] with synthetic dyes has extended the light-harvesting range of these complexes *in vitro*. Biohybrid approaches have led to augmented light harvesting by interfacing with non-native antenna complexes on inorganic surfaces [35], with silver electrodes that transfer plasmonic energy to RC-LH1-PufX [36] and mixing of RC-LH1-PufX complexes with different carotenoid content in solar cells [37]. Immobilised LH2 complexes on arrays of gold nanostructures exhibit strong coupling between the localised surface plasmon resonances and LH2 excitons, and a major new absorption feature was found at 649 nm [38].

**Fig. 2:**
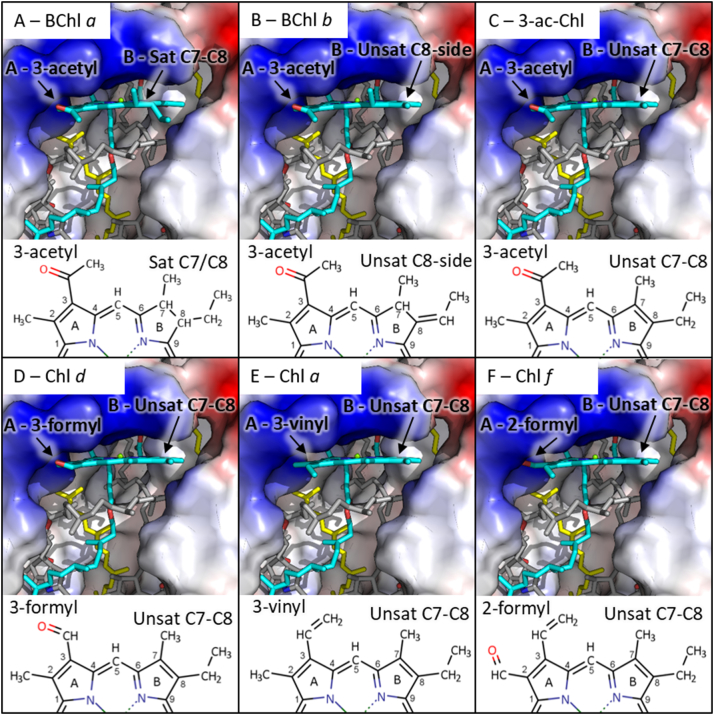
Models of (B)Chls in the B800 site of *Rbs*. *acidophilus* LH2 (PDB ID: 1NKZ) [14]. The polypeptides are shown in surface representation with positive charges in blue, negative in red and uncharged regions in white. Cofactors are in stick representation with B850 in grey and carotenoid in yellow. The B800 BChl *a* has been coloured cyan (Panel A). Models of LH2 containing BChl *b* (Panel B), 3-acetyl Chl *a* (Panel C), Chl *d* (panel D), Chl *a* (Panel E), and Chl *f* (Panel F) in the B800 site are also shown. Below each structure are 2D representations of the A and B rings of each (B)Chl with rings A, B and carbons 1–9 labelled.

Photosynthetic eukaryotes, cyanobacteria and other purple bacteria produce a wide range of (bacterio)chlorophylls ((B)Chls) that absorb light over a wide range of wavelengths. These pigments differ from BChl *a* by desaturation of the C7=C8 bond and their substitutions to the porphyrin side groups (Fig. 2). As the synthesis of (B)Chls requires a common precursor, Chlide [39], engineering of *Rba*. *sphaeroides* to produce alternative (B)Chls has been an inviting goal. Thus far, low levels of Chl *a* have been engineered in *Rba*. *sphaeroides*, whilst retaining native BChl *a* production [40,41]. BChls *b* and *g* have also been produced by redirecting the BChl *a* pathway [42,43]. If strains can be engineered to produce large quantities of these (B)Chls alongside the native BChl *a*, it is essential that they are incorporated into the light-harvesting antennas. The simplest method is to incorporate them into the native antenna complexes, which self-assemble into arrays optimised for the transfer of energy to the RC [2,3,[6], [7], [8], [9]].

As the pigment-protein complexes of purple bacteria have evolved to utilise BChl *a*, it is not surprising that the majority cannot accommodate a wide range of alternatives in reconstitution experiments. The accessory BChl *a* of the RC can be exchanged for modified variants, with detrimental effects on its photochemistry [[44], [45], [46]]. The LH1 complexes of *Rhodospirillum rubrum* are unable to bind Chl *a*, Chl *b*, 3-acetyl Chl *a*, and modified BChl *a*, but will accept BChl *b* [47]. Thus far there are no reports of incorporation of non-native pigments into the structurally similar B850 sites of LH2. The B800 site of LH2 is exceptional in that it can bind a wide range of (B)Chls, which makes this site a promising target for the integration of engineered non-native pigments. By chemical treatment, the B800 BChl *a* can be removed and replaced with alternatives [[48], [49], [50], [51], [52], [53], [54]]. Promisingly, Chl *a* has been shown to bind correctly [55] and experiences a similar microenvironment to the native BChl *a* [15]. Reconstituted (B)Chls efficiently absorb light and transfer energy to the native B850 BChl *a*, even if there is little or no spectral overlap between them [49,50,56].

Here, we expand upon these studies using *Rba*. *sphaeroides* LH2. We have systematically studied the specificity of the B800 site and find that it strongly favours BChl *a* over the other (B)Chls tested. Using both previously studied pigments alongside novel reconstitutions, we find that the saturation of the C7 and C8 bonds, and H-bonding to the 3-acetyl group contribute to this specificity. We then engineered the B800 site to reduce specificity, which results in a binding site that appears equally able to bind Chl *a* and BChl *a* whilst retaining efficient energy transfer to B850.

## 2 Materials and methods

### 2.1 Modelling of alternative (B)Chls in the B800 site

The *Rbs*. *acidophilus* LH2 structure was downloaded from the RSCB-PDB (PDB ID:1NKZ) [14]. The chain A B800 BChl *a* was edited using UCSF Chimera [57]. Where the C7 and C8 bonds were saturated, the geometry of the side-groups was made consistent with a trigonal planar molecular geometry. For the 3-formyl and 3-vinyl groups the bond angles were left unaltered. For Chl *f* the 2-methyl group was altered to formyl and the dihedral angle was adjusted to minimise the distance between the oxygen and the βArg_−10_ residue. Modified (B)Chls were incorporated into LH2 with the PyMOL Molecular Graphics System, Version 2.1 (Schrödinger, LLC) by alignment to the B800 BChl *a* molecules.

### 2.2 Generation of *Rba*. *sphaeroides* strains

*Rba*. *sphaeroides* strain Δ*pufBALMX* was generated by deleting the *pufBALMX* genes using pK18mobsacB as described previously [24,58]. Briefly, PCR primers were designed to amplify regions ~400 bp upstream (BALMX KO Upstream F and R) and downstream (BALMX KO Downstream F and R) of the genes encoding *pufBALMX*. These were designed with complementary overhangs on the upstream R and downstream F primers to allow fusion of the two amplified sequences by PCR. This generated a fragment of genomic sequence with genes encoding *pufBALMX* omitted. The upstream F primer contained an overhanging *Eco*RI site and the downstream R primer contained an overhanging *Hin*dIII site to allow restriction-ligation into the pK18mobsacB vector [59]. The resulting vector (pMobsacPufBALMX-KO) was mated into wild-type *Rba*. *sphaeroides* via conjugation with transformed *E*. *coli* S17-1 cells under kanamycin selection, then transferred to M22 agar [60] plates supplemented with sucrose. Successful integration was confirmed by loss of kanamycin resistance and colonies were screened by PCR using primers *pufBALMX* screen F and R. Successful gene deletion results in a reduction of the PCR product size. The mutation was then confirmed by DNA sequencing of a PCR amplicon encoding the modified *puf* operon.

The Δ*puc1BA* Δ*puc2BA* Δ*pufBALMX* strain was produced from an existing Δ*puc1BA* strain [61]. The *pufBALMX* genes were removed with the pMobsacPufBALMX-KO plasmid described above. *puc2BA* was deleted using the pMobsacPuc2BA-KO vector. This was generated by amplification of the wild-type *Rba*. *sphaeroides* genome by overlap PCR omitting the *puc2BA* coding region as described above with primers Puc2BA KO Upstream F and R, and Puc2BA KO Downstream F and R. Due to incompatibility with the target sequence, the *Eco*RI site was exchanged for *Xba*I in Puc2BA KO Upstream F. Successful mutations were detected by PCR with primers Puc2BA Screen F and R and confirmed by DNA sequencing of the *puc2BA* operon.

A plasmid containing *pucBA* with the βR30L substitution was generated by overlap PCR of the *pucBA* genes with ~400 bp upstream and downstream of the coding region. The overlapping regions of primers Puc1B R30L F and R were designed with a C to G substitution resulting in a PucB Arg_−10_ to Leu codon exchange. Terminal primers Puc1BA Upstream F and Puc1BA Downstream R included *Eco*RI and *Hin*dIII sites respectively, which were used to ligate the PCR product into the pK18mobsacB vector generating pMobSacBR30L. Using QuikChange Lightning Mutagenesis^@^ (Agilent), pMobSacBR30L was altered to encode Phe in *pufB* codon 30 by a C to G base change using primers R30F QuikChange F and R. The pMobSacBR30F and pMobSacBR30F vectors were used to restore the *puc1BA* genes in strain Δ*puc1BA* Δ*puc2BA* Δ*pufBALMX* as described above. Successful integration was confirmed by increase of the *puc1BA* PCR product size using primers Puc1BA screen F and R followed by DNA sequencing. All primer sequences utilised here are reported in Table.S1.

The strains expressing βR30E and βR30M LH2 were produced by mating of existing pRK415 expression vectors [18] into the Δ*puc1BA* Δ*puc2BA* Δ*pufBALMX* strain via conjugation with S17-1 *E*. *coli* as previously described [60].

### 2.3 Growth of bacterial strains

*Rba*. *sphaeroides* cells were grown in 1.6 L M22 medium supplemented with 1 g L^−1^ casamino acids [60] in 2 L flasks in the dark at 34 °C for 72 h shaking at 180 rpm. Strains harbouring pRK415 vectors were also supplemented with 5 μg mL^−1^ tetracycline. Cells were harvested by centrifugation at 4000 RCF. *Blastochloris (Blc.) viridis* cells were grown in 1 L Roux bottles illuminated by OSRAM 116 W halogen bulbs at 50 μmol s^−1^ m^−2^ at room temperature in *Rhodospirillaceae* medium (DSMZ medium # 27) [42] for 3 days whilst stirring and harvested at 4000 RCF for 30 min. *Synechocystis* sp. PCC 6803 was grown in 1 L BG-11 medium [62] with aeration under 100 μmol s^−1^ m^−2^ illumination from OSRAM CLASSIC 116 W halogen bulbs at room temperature for 5 days and harvested by centrifugation at 17700 RCF (avg) at 4 °C for 20 min. *Acaryochloris marina* cells were grown as described for *Synechocystis* sp. PCC 6803 with the addition of 32 g L^−1^ instant ocean (Aquarium systems) [63]. *Fischerella thermalis* PCC 7521 cells were grown as described in [64].

### 2.4 Purification of LH2 complexes

Cells were broken by two passes through a French pressure cell (Aminco, USA) at 20,000 psi. Insoluble material was removed by centrifugation at 18459 RCF (avg) for 15 min at 4 °C. Supernatants were loaded onto 40/15% (w/v) sucrose step gradients (in 20 mM Tris pH 8) and centrifuged at 57031 RCF (avg) for 10 h at 4 °C. Membrane bands were harvested from the 40/15% sucrose interface and solubilised in 20 mM Tris pH 8 containing 0.5% w/v lauryldimethylamine N-oxide (LDAO) for 1 h in the dark at room temperature whilst gently stirring. Solubilised membranes were loaded onto a 50 mL DEAE Sepharose column (GE Healthcare) equilibrated with Buffer A: 20 mM Tris pH 8, containing 0.1% w/v LDAO. The column was washed with two volumes of Buffer A, then with four volumes Buffer A containing 150 mM NaCl. LH2 was eluted over two volumes with a linear gradient from 150 to 250 mM NaCl. Fractions with the highest absorption ratios between 850 and 280 nm (A850/A280) were pooled, diluted three-fold and used to repeat the purification procedure twice. Fractions with A850/A280 above 2.5 were pooled, concentrated to 2 mL and loaded onto a Superdex 200 16/60 column (GE Healthcare) pre-equilibrated with 20 mM Tris pH 8 containing 200 mM NaCl and 0.03% w/v *n*-dodecyl-β-d-maltopyranoside (β-DDM) and eluted over 1 column volume of the same buffer. LH2 containing fractions with A850/280 above 2.8 were stored at −20 °C.

### 2.5 Selective removal of the B800 BChl *a*

B800 was removed by adaptation of the methods in [48,49]. As the detergent Triton BG-10 was not available, it was exchanged for lithium dodecyl sulphate (LDS), which has been shown to reversibly remove B800 in LH2 [15,52,65]. Purified LH2 complexes were incubated either in 20 mM sodium acetate pH 5 (WT) or 20 mM Tris pH 8 (βR30L and βR30F), containing 100 mM NaCl, 0.04% w/v β-DDM and 0.02% w/v LDS for 30 min at room temperature in the dark. Solutions were bound to a 50 mL DEAE column pre-equilibrated with the appropriate incubation buffer then washed with four column volumes of the same buffer followed by four volumes of 20 mM Tris pH 8 containing 100 mM NaCl and 0.03% w/v β-DDM. LH2 complexes were eluted by increasing the NaCl concentration to 300 mM. For WT LH2, if residual B800 was present in the absorption spectrum this procedure was repeated.

### 2.6 Preparation of (B)Chls

All (B)Chl preparations were performed in the dark to avoid photodamage of the pigments using an adaptation of the method in [66]. Chls *a*, *d* and *f* were extracted from cells with 35 mL methanol. Sodium D-ascorbate was added to a concentration of 10 mM (to protect against oxidation) followed by removal of cellular debris and proteins by centrifugation at 4700 RCF (avg) for 30 min at 4 °C. The supernatant was carefully separated from the pellet and 3.5 mL dioxane was added followed by 3.5 mL H_2_O, dropwise whilst stirring. The volume was reduced to 10 mL by evaporation under a stream of nitrogen gas. Precipitated chlorophylls were harvested by centrifugation at 4700 RCF (avg) for 30 min at 4 °C, pellets were thoroughly drained then dissolved in 2 mL methanol. For preparation of BChls from *Rba*. *sphaeroides* and *Blc. viridis*, the addition of dioxane and water were omitted.

For the preparation of Chl *f*, the extracted pigments were separated by injection onto a 5 μm UniverSil C18 150 × 10 mm column (Fortis) by HPLC and eluted at 3.5 mL min^−1^ with a linear gradient of 10% solvent A (30% v/v methanol + 350 mM ammonium acetate)/90% methanol to 100% methanol over 30 min [67]. Fractions containing Chl *f* were collected and pooled. For Chl *d* extractions, Chl *d* was >95% of the total (data not shown) and was not further purified. All other pigments are the sole (B)Chl produced by their respective species and did not require further purification.

3-acetyl-Chl *a* was produced by adaptation of the method in [68]. Purified BChl *a* was dissolved in acetone and 3,4,5,6-tetrachloro-1,2-benzoquinone was added in a 1:1 molar ratio. The solution was incubated at room temperature in the dark for 3 h. The 3-acetyl Chl *a* was enriched by HPLC as for Chl *f* (data not shown).

All chlorophylls were dried by vacuum centrifugation and stored at −20 °C. Extinction coefficients for determination of the Chl concentrations were: BChl *a*, 54.8 mM^−1^ cm^−1^ at 771 nm in methanol [69]; BChl *b*, 106 mM^−1^ cm^−1^ at 791 nm in diethyl ether [70]; Chl *f*, 78.36 mM^−1^ cm^−1^ at 707 nm in methanol [71]; Chl *d*, 71.11 mM^−1^ cm^−1^ at 697 nm in methanol [71]; 3-acetyl-Chl *a*, 65.2 mM^−1^ cm^−1^ at 677 nm in acetone [68]; and Chl *a*, 71.43 mM^−1^ cm^−1^ at 665 nm in methanol [72].

### 2.7 Reconstitution of B800 with alternative (bacterio)chlorophylls

B800-depleted LH2 was diluted to an absorption of 1 at 850 nm in 1 × 1 cm cuvette with 20 mM Tris pH 8, containing 0.5% w/v β-DDM and 12 μM of the required (B)Chl. Samples were prepared by dissolving the (B)Chl in methanol at a concentration of 1–2 mM. (B)Chl was added to 10% w/v β-DDM and diluted with the required quantity of 20 mM Tris pH 8 before adding B800-depleted LH2 to ensure the protein was not exposed to >2% v/v methanol. Solutions were incubated overnight at room temperature in the dark. Samples were concentrated to 1 mL and applied to a DEAE column equilibrated with 20 mM Tris pH 8, containing 0.03% w/v β-DDM. The column was washed with five volumes of 20 mM Tris pH 8 containing 100 mM NaCl and 0.03 w/v β-DDM. Reconstituted LH2 complexes were eluted by increasing the NaCl concentration to 300 mM.

### 2.8 Chlorophyll binding and competition kinetics

Binding kinetics were measured as described for the reconstitution of B800 with alternative (B)Chls in a 1 mL cuvette collecting spectra between 650 and 1000 nm over 180 or 540 min. For competition assays, as-prepared or B800-reconstituted LH2s were incubated. Kinetic traces were generated by plotting the ratio of absorption at 850 nm and of the pigment under investigation, and were fit to a single-exponential decay model using Graphpad Prism 7.

### 2.9 Spectroscopic methods

For absorptance and excitation measurements, LH2 complexes were diluted to an absorbance of 0.15 at 850 nm in 1 cm reduced volume quartz cuvettes. Absorption spectra were collected (Shimadzu UV-1800) and converted to absorptance (for comparison to excitation spectra) via Eq. (1):

(1) $$\mathrm{Absorptance}=1-10^{-\left(\mathrm{Absorbance}\right)}$$

3D fluorescence excitation spectra were collected on a Nanolog (Horiba) from 300 to 890 nm using 1 nm steps, a 3 nm bandpass (both excitation and emission), 4 s integration times, and monitoring LH2 B850 fluorescence at 890–900 nm. A tuneable bandpass emission filter (Semrock Versachrome TBP01-900/11, 0°) was used to remove any free BChl *a* fluorescence and stray excitation light.

Transient absorption experiments utilised LH2 with absorbance of 0.5 at 850 nm in 2 mm reduced volume quartz cuvettes whilst stirring. An amplified Ti:Sapphire laser system (Spectra Physics) and Helios spectrometer (Ultrafast Systems) provided ~0.2 μJ (800–910 nm detection region) or ~0.4 μJ (450–750 nm detection region), ~100 fs excitation pulses (at 1 kHz).

The 3-acetyl-Chl *a* was prepared as ~1 μM solutions in toluene and pyridine in 1 cm quartz cuvettes and the lifetimes obtained using time-correlated-single-photon-counting (TCSPC) detection of fluorescence decay (with an instrument response of ~0.2 ns) at 700 nm (following 690 nm excitation).

Efficiency of energy transfer from the lifetimes was calculated according to Eq. (2):

(2) $$\Phi_{\mathrm{EET}}=1-\frac{\tau_{\mathrm{LH}2}}{\tau_{\mathrm{solvent}}}$$

where *τ*_LH2_ and *τ*_solvent_ are the lifetimes of the (B)Chls reconstituted into LH2 and pure pigments dissolved in toluene or pyridine, respectively.

Fluorescence emission spectra were collected on a Fluorolog 2 (Horiba) fluorescence spectrophotometer equipped with a tungsten light source (OSRAM) and a Photocool series chilled PMT detector. Samples were diluted to an absorbance of 0.1 at 850 nm in either 20 mM Tris pH 8 containing 0.03% w/v β-DDM, or methanol. Fluorescence emission spectra were collected between 600 and 800 nm upon excitation at 435 nm (Chl *a* and 3-acetyl-Chl *a*) or 455 nm (Chl *d*). Data were collected at 0.5 nm wavelength intervals with a 0.25 s integration time and are the product of 32 summed scans. Energy transfer efficiency was estimated according to Eq. (3):

(3) $$\Phi_{\mathrm{EET}}=\frac{{\mathrm{Int}}_{s}}{{\mathrm{Int}}_{b}}$$

where Int_s_ and Int_b_ are the maximal fluorescence intensities for samples in methanol and 20 mM Tris/0.03% w/v β-DDM buffer, respectively.

All spectroscopic measurements were performed at room temperature.

## 3 Results

### 3.1 Modelling of (bacterio)chlorophylls in the B800 binding site suggests interactions with the A and B rings are important

To gain further insight into (B)Chl binding to the B800 site, we examined the crystal structure of *Rbs*. *acidophilus* LH2 [14]. This structure shares 45% and 66% sequence identity with the α and β polypeptides of *Rba*. *sphaeroides* LH2, respectively (data not shown) and key conserved residues are essential for correct binding of BChl *a* in the *Rba*. *sphaeroides* complex [17,20,73].

Fig. 2A shows the structure of the B800 site containing BChl *a*. An annotated 2D diagram of the BChl *a* A and B rings is shown below the structure. BChl *a* is bound with rings C, D and E in the protein interior along with the phytyl tail. Rings A and B are located at the periphery of the protein matrix with the ring B side groups extending into the external lipid or detergent environment. The areas above and adjacent to the tetrapyrrole ring form a large, positively charged region comprised of multiple residues, which include the H-bonding βArg_−10_ and the α-polypeptide C-terminal loop. Below the tetrapyrrole ring are the phytyl tails of the B850 BChl *a* molecules and the carotenoid, which both interact with the phytyl tail of the B800 BChl *a*.

The effects of C7 and C8 bond saturation, and substitution of the 3-acetyl group were examined by substituting the B800 BChl *a* *in silico*. Fig. 2B shows a model of the B800 site containing BChl *b*, a red-shifted BChl found in *Blc*. *viridis* [74], which differs from BChl *a* by an unsaturated ethylidene bond between the C8 carbon and its side group [75]. This bond alters the geometry of C8 to trigonal planar, bringing its side-group closer to the protein matrix above the binding pocket. Panel C shows a model of the B800 site containing 3-acetyl Chl *a*, a non-natural pigment that differs from BChl *a* by desaturation of the B-ring C7-C8 bond yielding a chlorin tetrapyrrole [68]. C7-C8 desaturation produces a rearrangement of the C8 side group to a geometry similar to that found in BChl *b* and reorients the C7 methyl group in-plane with the macrocycle and away from the protein matrix. Fig. 2D shows LH2 containing Chl *d*, a red-absorbing Chl found in some cyanobacteria such as *Acaryochloris* species [76]. Chl *d* contains an unsaturated C7=C8 bond in its B-ring, and has a formyl group as the C3 side-group in the A ring, which contains a potential H-bond accepting oxygen. Panel E shows a model containing Chl *a* in the B800 site, which has an unsaturated C7=C8 bond in the B ring and has a vinyl group as the A-ring C3 side-group that lacks an H-bond accepting oxygen. The final chlorophyll modelled is the recently discovered Chl *f* (Fig. 2F), the most red-shifted Chl discovered to date [77], with a B ring structure and the C3 side group identical to those of Chl *a*. However, the C2 formyl in Chl *f* places an oxygen in a different position to the 3-acetyl and 3-formyl (B)Chls modelled, and the ~3 Å distance between the 2-formyl oxygen and βArg_−10_ nitrogen allows a potential H-bond within this binding pocket.

### 3.2 The B800 of LH2 can be exchanged for Chl *a*, Chl *d*, 3-acetyl Chl *a*, and BChl *b*

**Fig. 3:**
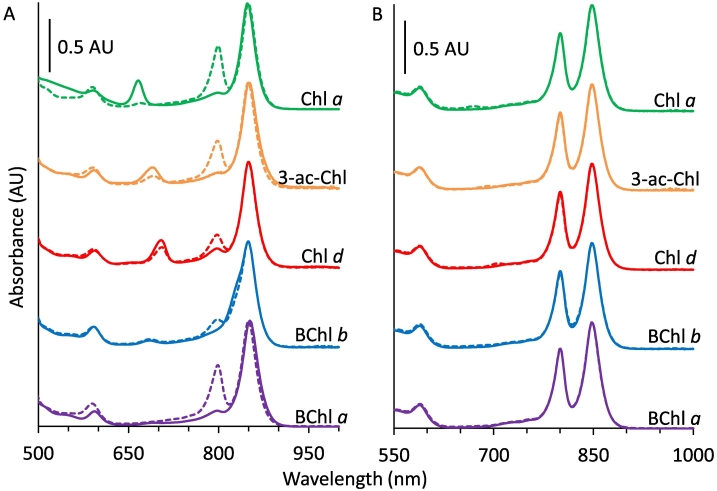
Panel A: Solid lines show absorption spectra of WT B850 LH2 (Purple), and WT B850 reconstituted with Chl *a* (green), 3-acetyl-Chl *a* (orange), Chl *d* (red) or BChl *b* (blue). Dashed lines show the spectra of the B800-substituted complexes following incubation with excess BChl *a* and removal of free pigment. Panel B: WT (B800–850) LH2 before (solid lines) and after (dashed lines) incubation with Chl *a* (green), 3-acetyl Chl *a* (orange), Chl *d* (red), BChl *b* (blue) and BChl *a* (purple).

To enable characterisation of (B)Chl binding to the B800 site of *Rba*. *sphaeroides* LH2, the native BChl *a* was removed by exchange into pH 5 buffer containing LDS. These complexes were termed B850 due to their sole 850 nm peak in the NIR region (Fig. 3A, solid purple line). B800 removal red-shifts the Q_y_ band of B850 by 3 nm and the Crt absorption by 5 nm as previously reported [48,49,52,73,78]. This effect is, in part, due to contraction of the LH2 ring altering the microenvironments and binding conformations of the pigments [15,79,80]. The vacant B800 sites readily bind a range of (B)Chls yielding complexes with new spectral features. We produced LH2 containing Chl *a* and 3-acetyl-Chl *a*, which have been described previously [15,49,[52], [53], [54]] (Fig. 3A, green and orange solid lines). We also produced novel complexes containing Chl *d* (recently incorporated into the analogous *Rbs*. *acidophilus* complex [81]) and BChl *b* (Fig. 3A red and blue solid lines). Upon (B)Chl binding the spectral properties of Crt and B850 are restored, and the newly incorporated (B)Chls contribute new absorption bands.

The rates of binding were measured by recording spectra during incubation of the B850 complexes with the non-native (B)Chls. The half-times for binding were 17.1 ± 0.3 min for BChl *a*, 6.7 ± 0.5 min for Chl *a*, 25 ± 3 min for 3-acetyl-Chl *a*, 22 ± 1 min for Chl *d* and 5.4 ± 0.4 min for BChl *b* (see Fig. S1 for raw data). These kinetics may not be a simple product of (B)Chl binding and do not appear to follow trends of relative binding strengths. As the (B)Chls are solubilised in detergent micelles before incubation with the protein, other processes may be rate-limiting and could differ for each pigment. Nevertheless, the kinetics show that (B)Chl binding is complete within a few hours, and overnight incubation is sufficient to ensure binding has progressed to completion.

### 3.3 Competitive binding assays reveal the specificity conferred by rings A and B of (bacterio)chlorophyll

The specificity of the B800 binding-site was examined by measuring the ability of the native BChl *a* to displace non-native (B)Chls that had been reconstituted into the B800 binding site of LH2. The results of competitive binding assays are reflected in the absorption spectra of B850 and (B)Chl reconstituted LH2 complexes (solid lines) in Fig. 3A. These complexes were then incubated in excess BChl *a* followed by removal of free pigments by ion-exchange chromatography (dashed lines). All LH2 complexes regained absorption at 800 nm, with concomitantly lowered absorption at the wavelength of the non-native (B)Chl, showing that BChl *a* displaces some of these pigments from the B800 site. In a reversed assay, as-prepared LH2 containing its native BChl *a* in the B800 site (i.e. not treated with LDS and reconstituted) was incubated in an excess of each non-native (B)Chl (Fig. 3B). Following incubation with BChl *a*, no spectral changes were observed. For all other (B)Chls almost no loss of the B800 band was observed with a negligible absorption increase corresponding to the exogenous pigment. Together, these data demonstrate that the B800 site is strongly selective for BChl *a* over the non-native (B)Chls.

The variable efficiency of BChl *a* binding to the reconstituted complexes is illustrated by the differing rise in absorption at 800 nm and corresponding loss of the reconstituted (B)Chl (Fig. 3A). By quantifying the change in absorption at 800 nm, an estimate of the relative binding strengths of these pigments can be made (Table 1). Additional insights can be obtained by analysing the rate of BChl *a* binding to the non-natively occupied B800 site. Plots of the change in absorption at 800 nm relative to 850 nm over a five-hour period are shown in Fig. S2 with half-times from single exponential fits in Table 1, which correlate well with the eventual increase in 800 nm absorption. The half-times in Table 1 in minutes reflect the replacement of a non-native pigment in the B800 site by the native BChl *a*. Release of the reconstituted (B)Chl is rate-limiting (compare with half-times of 5–17 min for initial reconstitution of (B)Chls mentioned above), and the clear differences seen for replacing Chl *d*, Chl *a* and 3-acetyl Chl *a* with BChl *a* reflect the varying tendencies of each pigment to occupy the B800 binding site.

**Table 1:** Change in (bacterio)chlorophyll absorption and binding rates for (bacterio)chlorophyll competition assays.

B800 (B)Chl	Free (B)Chl	λ monitored (nm)	ΔA (λ/850 ratio)	Half time (min)
BChl *b*	BChl *a*	800	0.087	230 ± 20
Chl *d*	BChl *a*	800	0.122	180 ± 10
Chl *a*	BChl *a*	800	0.276	115 ± 5
3-acetyl-Chl *a*	BChl *a*	800	0.295	69 ± 3
BChl *a* (WT)	BChl *b*	830	0.016	Inf^a^
Chl *d*	BChl *b*	830	0.137	150 ± 14
Chl *a*	BChl *b*	830	0.183	62 ± 3
3-acetyl-Chl *a*	BChl *b*	830	0.216	58 ± 3
BChl *a* (WT)	Chl *d*	705/800	0.024	Inf^a^
BChl *b*	Chl *d*	705/830	0.146	140 ± 20
3-acetyl-Chl *a*	Chl *d*	705/690	0.178	n/d^b^
Chl *a*	Chl *d*	705/665	0.227	47 ± 3^a^
BChl *a* (WT)	3-acetyl-Chl *a*	690	0.017	Inf^a^
Chl *d*	3-acetyl-Chl *a*	690	0.063	Inf^a^
BChl *b*	3-acetyl-Chl *a*	690	0.115	250 ± 70
Chl *a*	3-acetyl-Chl *a*	690	0.179	88 ± 4
BChl *a* (WT)	Chl *a*	666	0.026	Inf^a^
BChl *b*	Chl *a*	666	0.098	190 ± 30
Chl *d*	Chl *a*	666	0.084	120 ± 10
3-acetyl-Chl *a*	Chl *a*	666	0.119	100 ± 10

a Rate too slow or no observed changes to fit.

b Unable to generate reliable fit to data.

To assess the relative binding strengths further, the reconstituted LH2s were incubated in an excess of each (B)Chl. Absorption spectra before and after incubation are shown in Fig. S3 with their corresponding kinetic data in Figs. S4–S7. The data from these assays are summarised in Table 1. It is clear that all four of the reconstituted pigments readily undergo exchange, again highlighting the specificity for BChl *a*. Nevertheless BChl *b* and Chl *d* appear more competitive than Chl *a*. Surprisingly, 3-acetyl-Chl *a* is less competitive than the other 3-acetyl- and 3-formyl-(B)Chls. This may be due to degradation of the pigment during the assay or another unconsidered property that leads to a relatively limited binding within the B800 site.

### 3.4 Engineering the B800 site by removal of the hydrogen bond to the 3-acetyl group is sufficient for binding site promiscuity

**Fig. 4:**
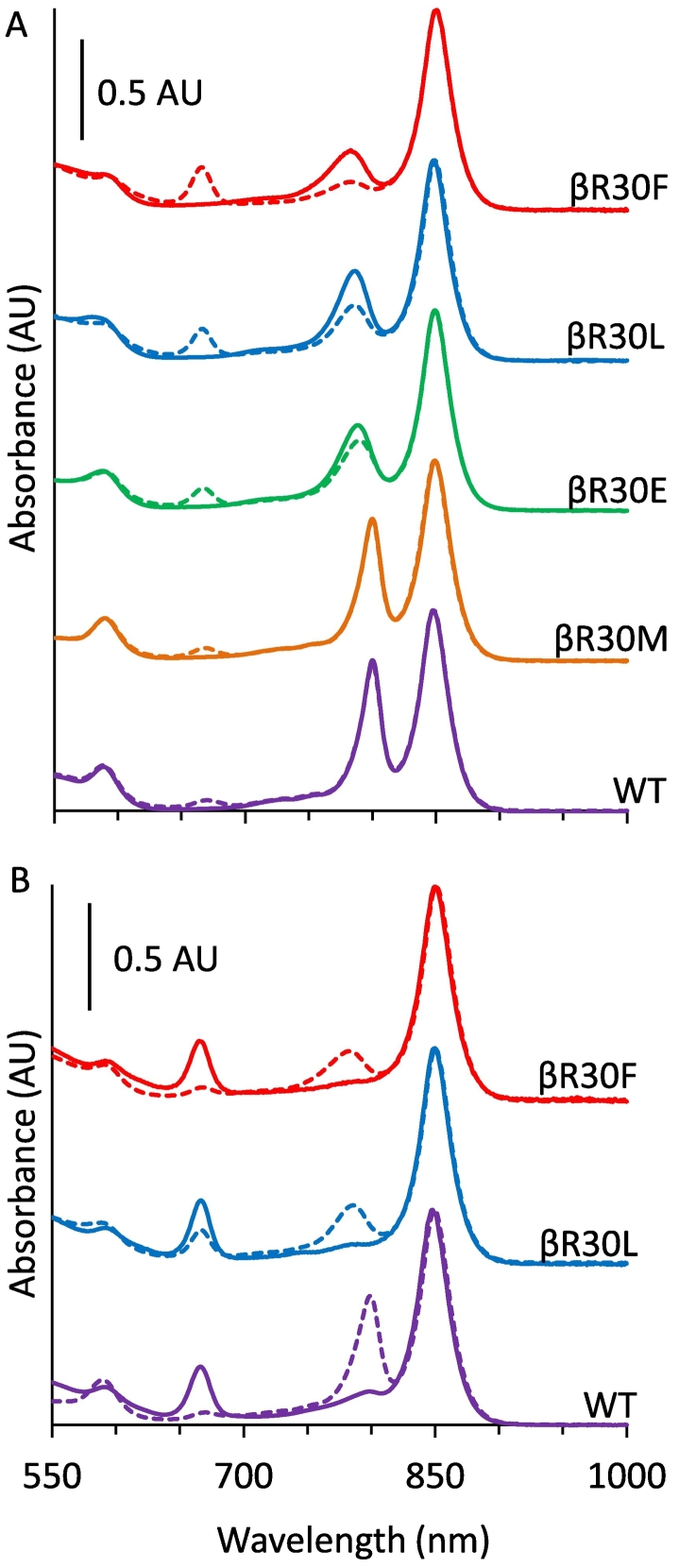
A. Vis/NIR absorption spectra of WT (purple), βR30M (orange) βR30E (green) βR30L (blue) and βR30F (red) LH2 complexes either containing B800 BChl *a* (solid lines) or following incubation in five-fold excess of Chl *a* (dashed lines). B: Absorption of spectra of WT (purple), βR30L (blue) and βR30F (red) LH2 containing Chl *a* in the B800 site (solid lines) or following incubation in excess BChl *a* (dashed lines).

We explored the possibility that the H-bond to the 3-acetyl group contributes to BChl *a* specificity by altering the βArg_−10_ residue, already known to form a H-bond to the B800 BChl *a* [18]. Deletion of the second LH2 operon (*puc2BA*) ensured that all LH2 β-subunits, encoded in the first operon *puc1BA*, were of the engineered sequence. In an initial screen four genomically encoded substitutions were tested and their absorption spectra are shown in Fig. 4A (solid lines). The βR30M substitution gives spectra identical to the wild-type (WT) with the B800 band at 800 nm and a FWHM of 20 nm. Previous reports suggest that this substitution provides an alternative H-bond donor for the 3-acetyl group of BChl *a* [19]. The second substitution, βR30L, has been shown to abolish H-bonding, resulting in a 14-nm blue-shift and attenuation of the B800 band to 786 nm with a FWHM of 32 nm [18]. Third was the βR30E substitution, which both abolishes the 3-acetyl H-bond and introduces a negative-charge in place of the native positively charged side chain, which results in a blue-shifted and attenuated absorption band at 789 nm with a FWHM of 34 nm [19]. Finally we generated a novel substitution, βR30F, to introduce a large-hydrophobic side-chain in the vicinity of the 3-acetyl group. This substitution blue-shifts the absorption band to 783 nm with a FWHM of 33 nm and attenuates it further than for βR30L. It should be noted that the βR30F complexes appeared to lose B800 BChl *a* during preparation, which could be restored by incubation in excess BChl *a* (Fig. S8). We therefore used βR30F that had been fully reconstituted with BChl *a* at the outset to ensure vacant B800 sites did not interfere with its characterisation. This treatment was not required for the other complexes, which were used as-prepared.

The mutant LH2 complexes fully occupied with BChl *a* in the B800 site were incubated in an excess of Chl *a* to examine its capacity to displace the native pigment. As shown in Fig. 4A with dashed lines, the greatest level of Chl *a* incorporation was observed for βR30F complexes, which showed a near complete loss of B800 and the presence of a new 666 nm absorption band, followed by βR30L and βR30E. Like the WT complex, βR30M showed little evidence of Chl *a* binding. Following this screen βR30L and βR30F were selected for further characterisation.

The reciprocal experiment involved adding back BChl *a* to Chl *a*-containing LH2 complexes to determine whether B800 BChl *a* specificity has been affected by replacement of βArg_−10_. This experiment required prior formation of B800-minus LH2 complexes, i.e. B850-only, so that as much Chl *a* as possible could be incorporated. Thus, βR30L and βR30F B850-only complexes were prepared at pH 8.0 (Fig. S9). The WT LH2 complex shows no loss of B800 when incubated in LDS at pH 8 (Fig. S9) so pH 5 was used, as described above (Fig. 3A, solid purple line). βR30L, βR30F and WT B850-only LH2 complexes were incubated in excess Chl *a*, and weakly bound or free Chl *a* was removed by ion exchange chromatography (Fig. 4B, solid lines). These complexes had almost indistinguishable Chl *a* bands centred at 666 nm with FWHM of 21 nm for WT and βR30F, and 19 nm for βR30L. The maximally reconstituted Chl *a*-B850 LH2 complexes were incubated with excess BChl *a* and then repurified, yielding the complexes in Fig. 4B (dashed lines). In each case there was an increase in absorption at 800 nm and a corresponding loss at 666 nm (Fig. 4B, dashed lines). Exchange is almost complete for the WT with very little Chl *a* absorption remaining at 666 nm; a similar situation occurs for the βR30F complexes. Intriguingly, significant Chl *a* absorption is retained for the βR30L complexes suggesting that exchange was significantly less efficient and the site has less discrimination for its native pigment.

**Fig. 5:**
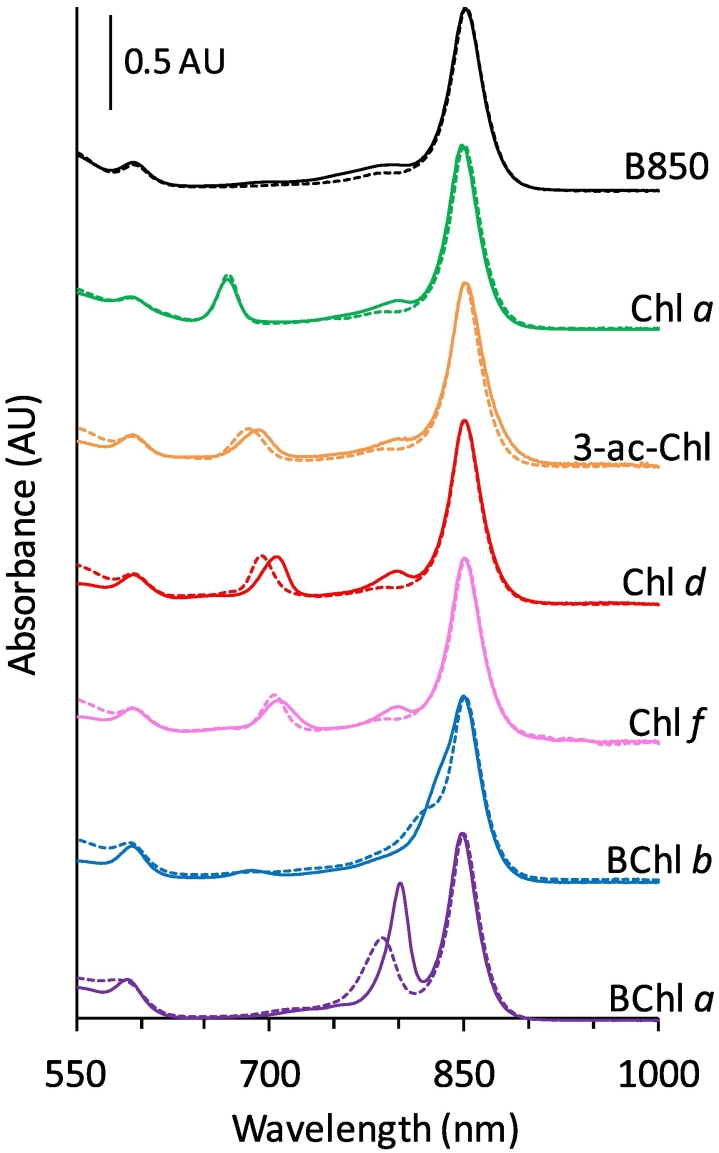
Absorption spectra of WT (solid lines) and βR30L (dashed lines) B850 LH2 complexes (black), and B850 complexes reconstituted with Chl *a* (green), 3-acetyl-Chl *a* (orange), Chl *d* (red), Chl *f* (pink) and BChl *b* (blue). Purple lines show as-prepared BChl *a* containing complexes.

As 3-acetyl-Chl *a*, Chl *d* and BChl *b* are expected to H-bond to the βArg_−10_ residue, we sought to assess this characteristic biochemically using the βR30L complexes that lack a H-bond donor to the 3-acetyl group. βR30L-B850 and WT-B850 complexes (Fig. 5 black dashed and solid lines, respectively) were reconstituted with Chl *a*, 3-acetyl-Chl *a*, Chl *d*, and BChl *b* (Fig. 5 dashed green, orange, red, and blue lines, respectively). As described above, βR30L and WT complexes reconstituted with Chl *a* have similar absorption spectra (Fig. 5, green lines). In contrast, 3-acetyl Chl *a*, Chl *d*, Chl *f* and BChl *b* show clear evidence of red-shifting when the native βArg_−10_ is present. The 3-acetyl-Chl *a* peak is located at 682 nm in βR30L and 691 nm in the WT suggesting the βArg_−10_ residue, and consequent H-bond, induces a 9-nm red-shift. The FWHM were similar at 32 and 35 nm for βR30L and WT, respectively. With Chl *d*, there is a 12 nm red-shift from 692 to 704 nm with FWHM of 23 and 27 nm. For BChl *b* the peak shifts from 817 to 829 nm for βR30L and WT, respectively. The FWHM for BChl *b* were not determined due to overlap with the B850 band.

As a formyl group at the 2-position contains an H-bond acceptor at a different location to that of 3-acetyl/formyl-(B)Chls, we generated additional novel reconstitutions using Chl *f* (Fig. 5). Interestingly, Chl *f* showed evidence of red-shifting when βArg_−10_ is present, albeit to a lesser extent than the 3-acetyl/formyl pigments at 4 nm. The peak is also broader in the WT LH2 with a FWHM of 37 nm than in βR30L with a FWHM of 27 nm. Due to the limited pigment with which to prepare the Chl *f* reconstituted complexes, they were not included in the competitive binding studies described above.

### 3.5 Energy transfer studies show reconstituted and engineered complexes are functional for energy transfer

**Fig. 6:**
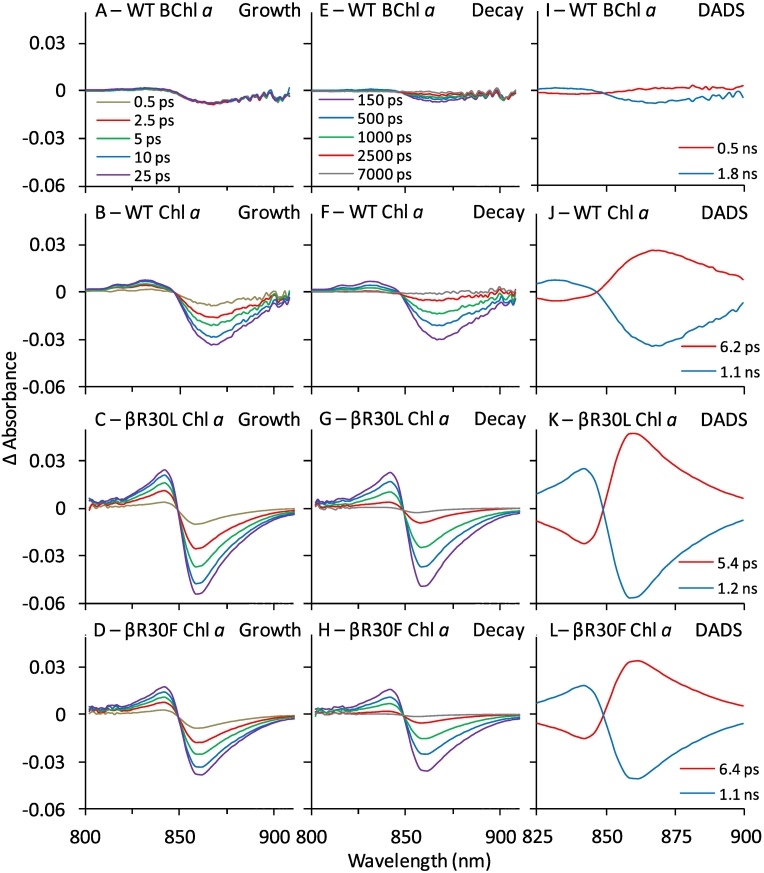
A–H: NIR TA spectra for select Chl *a*-containing samples (and related controls) obtained at indicated times (indicated in Panels A and E) after excitation with a 100 fs pulse at 665 nm. Spectra were normalised to absorbance of B850 band. Panels A–D show growth of B850* and panels E–H the decay of B800*. Panels I-L show DADS obtained from global analysis of the data sets. The DADS in red (6–10 ps) in J–L reflect decay of Chl a* (primarily energy transfer to B850) and the DADS in blue (~1 ns) in (I–K) is for decay of B850* to the ground state.

To determine whether the non-native (B)Chls in the B800 site are capable of efficient energy transfer to B850, reconstituted complexes were examined by transient absorption (TA) and steady-state fluorescence spectroscopies. A key indicator of energy transfer is a growth of bleaching of the B850 Q_y_ band in NIR TA spectra after specific excitation of the (B)Chl bound to the B800 site. When WT LH2 containing its native BChl *a* is excited at 665 nm, a small B850 Q_y_ bleach is observed due to the weak absorption at this wavelength (Fig. 6A). Near-identical signals and lifetimes were observed for the βR30L and βR30F BChl *a* containing complexes (Fig. S10). When the B800 site is reconstituted with Chl *a*, strong bleaching of B850 develops over the first several picoseconds and decays with a time constant of ~1 ns (Fig. 6B and F). This indicates that the energy absorbed by Chl *a* is transferred rapidly and efficiently to B850. For the Chl *a* reconstituted βR30L and βR30F complexes, a strong signal is also observed with similar rise and decay times (Fig. 6C, D, G and H) showing they are equivalent to the Chl *a* reconstituted WT.

Global analysis of each TA data set was performed to obtain best-fit time constants for the kinetic components and corresponding decay-associated difference spectra (DADS). The DADS will show a positive feature at 850 nm if that kinetic component reflects a growth of B850 bleaching (and stimulated emission) as a result of energy transfer, and will show a negative feature at that wavelength reflecting decay of excited B850 (B850*) to the ground state. The DADS for WT (Fig. 6I) shows only a weak negative feature at 850 nm due to decay of B850* because the absorption at 665 nm is small and does not appreciably excite the B800 BChls. However, upon excitation at 800 nm, the DADS shows a 0.5 ps component that reflects the lifetime of B800*, which decays by energy transfer to B850 (Table 2 and Fig. S11). Strong bleaching is also observed for BChl *a* containing βR30L and βR30F when excited at their corresponding Q_y_ maxima, with only a slight reduction in B800 lifetime as previously observed for βArg_−10_ substitutions [18] (Table 2 and Fig. S11). The DADS for WT, βR30L and βR30F containing Chl *a* reveal that the B850 bleaching grows more slowly, with time constants between 2 and 10 ps (Fig. 6J–L, red lines), which reflect the Chl a* lifetime. The global analysis does not show significant improvement if two B850* growth (Chl a* decay) components are used, returning an amplitude weighted lifetime similar to the analysis with one rise component (see Table S2).

**Table 2:** (B)Chl lifetimes bound in the B800 position in LH2^a^ or free in solvents^b^, and the associated energy transfer yield^c^. Energy transfer yields obtained from excitation-absorptance spectra and quenching are also included for comparison.

B800 Pigment	*τ*_LH2_^a^ (ps)	*τ* solvent (ps)^b^	Φ_EET_ (*τ*)^c^	Φ_EET_ (exc)^d^	Φ_EET_ (Quench)^e^	Φ_EET_ Average
Chl *a*	6.7 ± 0.7 (WT) 5.6 ± 0.4 (βR30F) 6.9 ± 0.5 (βR30L)	6300	>0.99 >0.99 >0.99	0.78 0.64 0.73	0.95 0.91 0.96	0.91 ± 0.12 0.85 ± 0.18 0.89 ± 0 0.15
3-Acetyl-Chl *a*	5.1 ± 0.5	5600	>0.99	0.88	0.89	0.92 ± 0.07
Chl *d*	3.5 ± 0.7	6500	>0.99	0.81	0.93	0.91 ± 0.10
Chl *f*	2.7 ± 0.8		>0.99	0.73	n/d^f^	0.9 ± 0.2
BChl *a*	0.5 ± 0.2 (WT) 0.8 ± 0.6 (βR30F) 0.6 ± 0.1 (βR30L)	3000	>0.99 >0.99 >0.99	1.03 0.97 0.94	n/d^f^	1.02 ± 0.02 0.99 ± 0.02 0.97 ± 0.04
BChl *b*	≤0.1^g^	2400	>0.99	1.05	n/d^f^	1.03 ± 0.04

a See Table S2 for component rates.

b Average of lifetimes obtained in pyridine and toluene (error ± 10%). See Table S3.

c Calculated using the formula Φ_EET_ = 1 − τ_LH2_/τ_solvent_.

d Obtained from the ratio of the integrated (B)Chl Q band in the excitation vs. absorptance spectra.

e Obtained by relative intensity of Chl *a* emission when bound to LH2 vs. free in solvent.

f Not determined due to overlap of B800 pigment spectrum and that of B850 BChl *a* or limited sample.

g Limited by the response of the instrument.

**Fig. 7:**
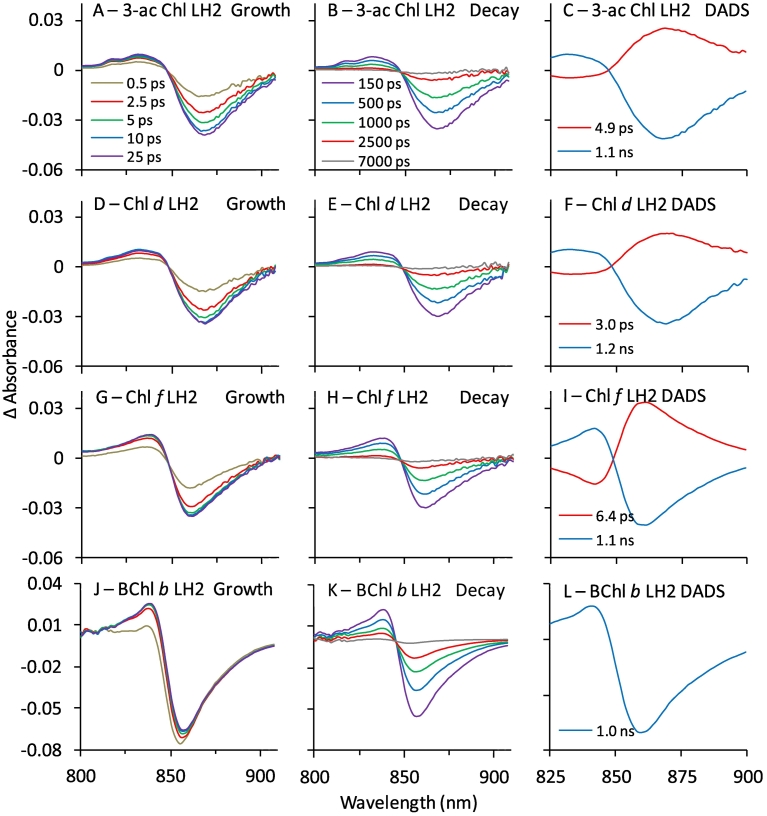
NIR TA spectra during growth or B850* (left panels) and decay of B850* (centre panels), and DADS (right panels) for WT LH2 reconstituted with 3-acetyl Chl *a* (Panels A–C), Chl *d* (D–F), Chl *f* (G–I), and BChl *b* (J–L). Samples were excited at 690 (3-acetyl Chl *a*), 700 (Chl *d* and Chl *f*), and 820 nm (BChl *b*).

The decay time of B850* is similar for all complexes at ~1 ns (Fig. 6J–L, blue lines), indicating that the properties of B850 were not perturbed by exchange of the B800 BChl *a* for Chl *a* and/or substitution of the βArg_−10_ residue. Similar TA experiments were performed on WT LH2 reconstituted with 3-acetyl Chl *a*, Chl *d*, Chl *f*, or BChl *b*. Strong bleaching of B850 developed over the first 10 ps or so in LH2 complexes reconstituted with (B)Chls when they were directly excited at their corresponding peak absorption wavelength (Fig. 7). The signals were much weaker in control experiments with BChl *a* containing complexes excited at these wavelengths, analogous to Fig. 6A (Fig. S12).

Analysis of the DADS and (B)Chl excited state lifetimes reveal that energy transfer from the (B)Chl in the B800 site increases by about two orders of magnitude as the B800 (B)Chl becomes more red-shifted. The time constant decreases from 6.7 ps for Chl *a* to <0.1 ps for BChl *b* (Fig. 7, summarised in Table 2). The <0.1 ps energy transfer time for BChl *b* containing complexes follows from the observation of no short-lived component to the TA data, indicating that energy transfer occurs within the 0.1 ps instrument response time. This finding likely reflects the significant overlap of the donor BChl *b* emission at 840 nm and B850 BChl *a* absorption, and follows the general trend in lifetime reduction as a function of wavelength derived from the other (B)Chls.

Comparison of the lifetimes of the excited (B)Chls when bound to LH2 versus in organic solvent (where energy transfer does not occur) allows energy-transfer efficiency (ΦEET) and rate constants (kEET) to be estimated. The lifetimes of the (B)Chls when bound to LH2 are 1000 to 10,000 fold faster than their lifetimes in solvent (Table 2 and Table S3). The dramatic enhancement when bound to LH2 indicates rapid and efficient energy transfer from the B800 (B)Chl to B850 BChl *a*. This is true for both excited-state lifetimes obtained from dual-exponential fits to the (B)Chl* decays (Table S2), indicating that some potential heterogeneity in the binding site for the non-native pigment does not diminish energy transfer efficiency. Comparison of the lifetimes of bound vs. free as described in the methods (Table 2, columns 2–3) returned energy-transfer yields approaching unity (Table 2, column 4).

**Fig. 8:**
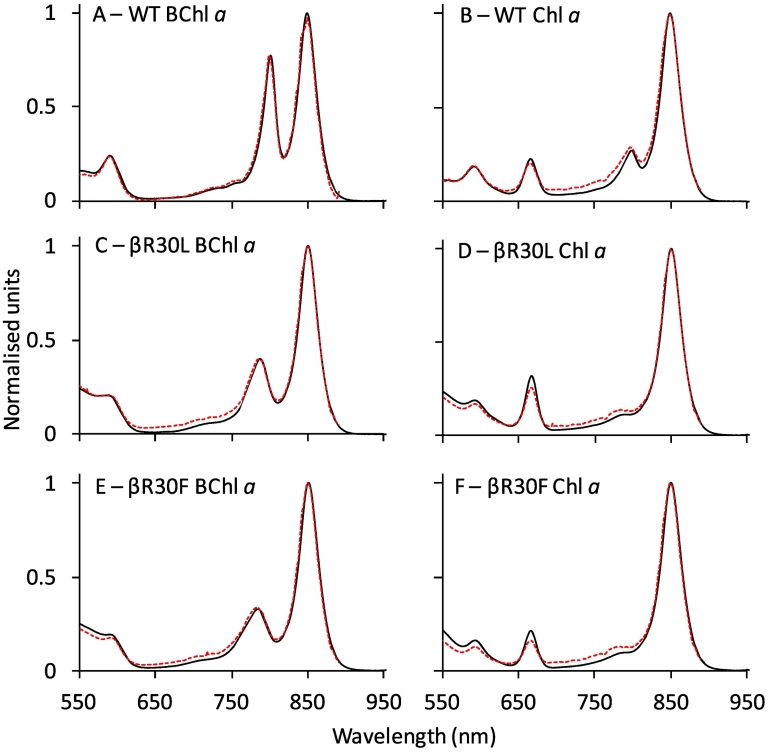
Absorptance (1 – transmittance) (black) and fluorescence-excitation (red) spectra of (A) WT LH2 containing B800 BChl *a*, (B) WT LH2 containing B800 Chl *a*, (C) βR30L LH2 containing B800 BChl *a*, (D) βR30L LH2 containing B800 Chl *a*, (E) βR30F LH2 containing B800 BChl *a*, and (F) βR30F LH2 containing B800 Chl *a*. Excitation spectra were obtained using fluorescence detection between 890 and 900 nm. All spectra are normalised at 850 nm.

Comparison of the absorptance (1 – transmittance) and fluorescence excitation spectra is another means of calculating energy transfer yields. The appearance of the non-native (B)Chl bands in the fluorescence excitation spectra (monitoring emission from B850) indicates effective energy transfer from the reconstituted (B)Chls (Fig. 8 and Fig. S13). Normalisation to the B850 band allows the relative efficiency of energy transfer across the spectrum to be assessed. Wavelengths at which the spectra overlay indicate a unity energy transfer efficiency, such as the B800 band in native WT LH2 (Fig. 8A), which is known to proceed with 100% efficiency [82]. Comparison of the Chl *a* band in reconstituted WT at 666 nm shows slightly lower intensity for the excitation spectrum than the absorptance spectrum (Fig. 8B) providing an estimate for Φ_EET_ of 78%. This suggests that a small amount of energy is lost by other means, such as fluorescence, internal conversion or intersystem crossing.

The BChl *a* containing βR30L and βR30F provide B800 to B850 energy transfer yields of 94% and 97%, respectively showing little to no loss of efficiency despite the blue-shift and attenuation of absorption (Fig. 8C and E and Table 2). When reconstituted with Chl *a*, the βR30L and βR30F complexes provide energy transfer yields of 73% and 64% respectively, similar to reconstituted WT. Estimates of energy transfer from the other (B)Chls in WT gave yields ranging in ~65–100% (Table 2, column 5 and Fig. S14) again showing energy transfer from all reconstituted (B)Chls is efficient.

A third estimate of energy transfer efficiency was obtained by comparing the fluorescence intensity of the LH2 complexes when native in buffer and with pigments free in solvent. The reduction in signal due to quenching of the pigment emission is a result of energy transfer to B850. As shown in Fig. S14 and summarised in Table 2 column 6, the estimates by this method range from 91 to 96%, in good agreement with the other two measurements. It should be noted that this method could not be employed for BChl *a* and BChl *b* due to the inability to discriminate between the spectra of the B800 pigment and the B850 BChl *a*, or for Chl *f* due to sample limitation.

**Fig. 9:**
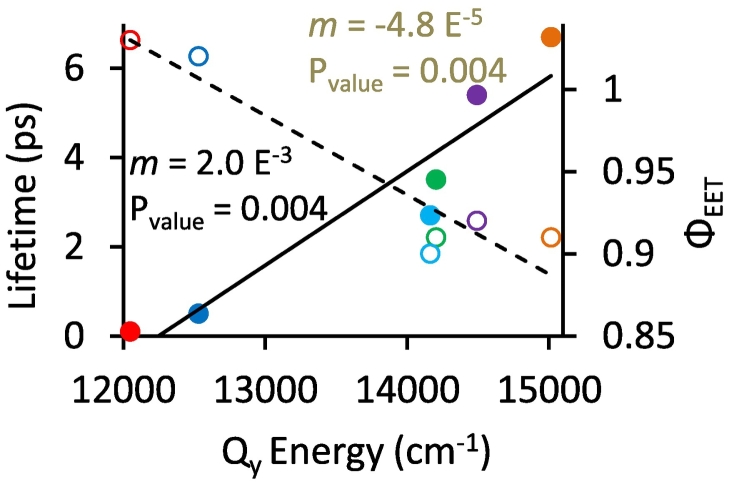
Trends in (B)Chl lifetime (black line and filled symbols) and energy transfer efficiency (Φ_EET_, dashed line and open symbols) as related to Q_y_ energy. The symbols representing the (B)Chls are as follows: BChl *b*-LH2 (red), BChl *a*-LH2 (WT, blue), Chl *f*-LH2 (cyan), Chl *d*-LH2 (green), 3-acetyl Chl *a*-LH2 (purple), and Chl *a*-LH2 (orange).

Taken together, the estimates above provide average energy transfer efficiencies from the reconstituted (B)Chls to B850 between 90 and 100% (Table 2, column 6). They also show that the energy transfer efficiency correlates with the absorption of the B800 (B)Chl becoming more efficient as Q_y_ absorption is further red-shifted. A similar trend was suggested in reconstituted *Rbs*. *acidophilus* complexes in which Chl *a* energy transfer is slightly less efficient than for Chl *d* or the native BChl *a* [81]. In our LH2 complexes we also observed a relationship for the energy transfer rate where transfer from B800 to B850 is accelerated as the B800 absorption band approaches that of B850 (Fig. 9).

## 4 Discussion

We have systematically assessed the B800 binding site specificity in *Rba*. *sphaeroides* LH2, advancing previous work showing this site could accommodate alternative (B)Chls [49], albeit at the expense of absorption at 800 nm. We rationalised this specificity by assessing the contributions of the 3-acetyl H-bond and B-ring C7–C8 bond saturation along with work by others suggesting the C, D and E ring structures are also significant [53,54]. Using these findings, we engineered LH2 to lower B800 site specificity producing a promiscuous pigment binding site.

Based on the B800 site modelling, it is apparent that alteration of the C, D and E rings is likely to have significant steric effects on binding. This prediction is supported by the work of Yoshitaka et al. who found that 3-acetyl-pyrochlorophyll is less efficiently incorporated into the B800 site of *Rbs*. *acidophilus* LH2 than 3-acetyl Chl *a*. This pigment differs from 3-acetyl-Chl *a* in that the C17-C18 bond of the D-ring is unsaturated [53]. Additionally, pyrochlorophyll *a*, which has a hydrogen as the E-ring C13^2^ side-group in place of a methoxycarbonyl group, is less efficiently incorporated than Chl *a* [54]. On the other hand, the A and B rings appear to have a larger degree of conformational freedom. Therefore, the pigments selected for this study have identical C, D and E rings with differences only in rings A and B, as is the case for the non-native (B)Chls produced in *Rba*. *sphaeroides* to date [[40], [41], [42], [43]].

Reconstitution of WT and R30L complexes shows that (B)Chls with 3-acetyl and 3-formyl groups form H-bonds to the βArg_−10_ residue inducing spectral red-shifts of 9–15 nm. This suggests that the effects of this interaction on the electronic structure are similar for BChl *a*, BChl *b*, Chl *d* and 3-acetyl-Chl *a* indicating similar rotation of the 3-acetyl/formyl dihedral angle. Our findings are paralleled by Saga et al. who used Raman spectroscopy to demonstrate a βArg_−10_ H-bond in *Rbs*. *acidophilus* complexes reconstituted with Chl *d* [81], as predicted in our model in Fig. 2. The spectral differences in Chl *f*-reconstituted complexes were less pronounced. As the predicted H-bond distance for Chl *f* is longer by around 1 Å than for (B)Chls with H-bond acceptors in the 3-position, it is expected to be substantially weaker. Additionally, the effect of H-bonding on the dihedral angle of the 2-group is likely to differ from pigments that H-bond at the 3-group, which will result in differences in the perturbation of the electronic structure. Nevertheless, this result suggests that Chl *f* may form an H-bond to the βArg_−10_ residue, augmenting its binding and spectral properties. Chl *a* does not have an H-bond acceptor in the 2- or 3-position and the wavelength of the Q_y_ band is almost identical in WT, βR30L and βR30F complexes at 666 nm, suggesting that the presence of βArg_−10_ has no influence on its electronic structure when bound to the B800 site.

The 3-acetyl H-bond to βArg_−10_ is thought to contribute considerably to binding energy [83]. In B875 of LH1 an H-bond to Trp contributes 3.7 kcal mol^−1^ [84]. The potential for H-bonding would suggest that BChl *a*, BChl *b*, Chl *d* and 3-acetyl Chl *a* should bind preferentially to the B800 site over Chl *a*. In agreement with this notion, BChl *b* and Chl *d* show higher relative binding strengths than Chl *a* under the experimental conditions tested. Despite the influence of the 3-acetyl H-bond the reconstituted pigments capable of forming this interaction cannot out-compete BChl *a*, suggesting the B-ring structure also contributes to B800 site specificity. Data from the modelling imply that alteration of the B-ring C7 and C8 bond saturation alters the geometry of their side groups and their proximity to the protein matrix. Taken together, these data show that both the A and B-ring structures influence the B800 binding site specificity. These findings are at odds with a recent publication that suggested that 3-acetyl Chl *a* binding may be slightly stronger than that of BChl *a* [53]. The discrepancy may be the result of inherent differences between the *Rba*. *sphaeroides* and *Rbs*. *acidophilus* LH2, or a result of the different competitive reconstitution methods used.

To lower the specificity of the B800 binding site we engineered four LH2 variants where βArg_−10_ was replaced with alternative residues. Our findings suggest that residues unable to form H-bonds to the 3-acetyl group are sufficient to abolish the strong BChl *a* specificity enabling effective displacement of BChl *a* by Chl *a*. The spectra of the engineered LH2s reconstituted with Chl *a* show enhanced occupancy relative to the Chl *a* reconstituted WT.

In order for these LH2 complexes to be viable for light-harvesting in bacterial strains that produce multiple Chls the non-native B800 (B)Chl must effectively transfer energy to the B850 BChl *a*, from which subsequent energy transfer to LH1 and RCs may occur. Spectroscopic analysis of the reconstituted LH2 complexes reveals that energy transfer remains highly efficient despite slowing of the energy transfer rate up to 10-fold, showing that the engineered Chl *a*-containing complexes could be viable as a light harvesting antenna. For 3-acetyl Chl *a* and Chl *a* in WT LH2 the rates are similar to *Rbs*. *acidophilus* LH2 complexes where B800 was reconstituted with the same pigments, and a similar dependence for the B800 to B850 energy transfer rate on wavelength was observed [50]. The finding that Chl *d* and Chl *f* have faster energy transfer rates than 3-acetyl-Chl *a*, owing to their absorption further into the far-red, is novel as is the finding that BChl *b* provides an enhanced energy-transfer rate when compared to the native BChl *a*-containing WT. Together these findings show that the energy transfer properties of *Rba*. *sphaeroides* and *Rbs*. *acidophilus* LH2 harbouring non-native pigments in their B800 sites are similar. They also show that spectral overlap is the key determinant of the energy-transfer rate from B800 to B850, with the possibility of exceeding that of the native complex by red-shift of the B800 band beyond 800 nm. A similar effect was previously achieved by blue-shifting of the B850 band [85]. Examination of the B850 excited-state lifetime revealed that all complexes, regardless of the pigment bound at the B800 site or substitution of the βArg_−10_ residue, had similar decay times of around 1 ns. This indicates that the properties of the B850 BChl *a* have not been altered by the reconstitutions or engineering of the B800 binding site further demonstrating their viability as an *in vivo* antenna.

Taken together, the work outlined here shows that LH2 complexes harbouring mixed Chl species are viable for harvesting light at wavelengths within the red-gap, which provides a route to enhancement of light-harvesting by LH2. The limitation of strong BChl *a* specificity of the B800 binding site can be overcome by removal of the H-bond to the 3-acetyl group of BChl *a*, generating a promiscuous binding site that readily binds Chl *a*. This has no negative impact on the spectroscopic properties and energy transfer from B800 to B850 demonstrating that the engineered LH2 complexes have the desired properties of promiscuous pigment binding whist retaining the ability for efficient light-harvesting.

Efforts to achieve the dual-synthesis of BChl *a* and Chl *a* *in vivo* in *Rba*. *sphaeroides* are ongoing in several laboratories. By combining this with the B800 binding-site engineering described here a promising route toward generation of strains of *Rba*. *sphaeroides* with enhanced light harvesting capabilities via simultaneous utilisation of multiple (B)Chls may be realised.

## Abbreviations

Rba. sphaeroides: *Rhodobacter sphaeroides*
LH2: light-harvesting complex two
RC-LH1-PufX: reaction-centre light-harvesting complex containing one PufX polypeptide
BChl a: bacteriochlorophyll *a*
Rbs. acidophilus: *Rhodoblastus acidophilus*
H-bond: hydrogen-bond
NIR: near infra-red
Chl: chlorophyll
LDS: lithium dodecyl sulphate
WT: wild-type
FWHM: full-width at half-maximum
NIR-TA: near infra-red transient absorption
ΦEET: energy transfer efficiency
kEET: energy transfer rate constant
DADS: decay associated difference spectra
LDAO: lauryldimethylamine N-oxide
β-DDM: *n*-dodecyl-β-d-maltopyranoside

## Transparency document

Transparency document. Image 1

## Appendix A Supplementary data

Supplementary material Image 2
