# Supplementary material for: Engineering of B800 bacteriochlorophyll binding site specificity in the Rhodobacter sphaeroides LH2 antenna
Source: Biochim Biophys Acta Bioenerg. 2019 Mar 1;1860(3):209–23. doi: 10.1016/j.bbabio.2018.11.008 (PMC6358721; doi:10.1016/j.bbabio.2018.11.008)
Supplement: Supplementary file 2 — Supplementary material [file mmc2.docx]

Supporting information for: Engineering of B800 bacteriochlorophyll binding site specificity in *Rhodobacter sphaeroides* LH2

**David J. K. Swainsbury^1^, Kaitlyn M. Faries^2^, Dariusz M. Niedzwiedzki^2^,**

**Elizabeth C. Martin^1^, Adam J. Finders^1^, Daniel P. Canniffe^1,3^, Gaozhong Shen^3^,**

**Donald A. Bryant^3^, Christine Kirmaier^2^, Dewey Holten^2^, and C. Neil Hunter^1^***

From ^1^Department of Molecular Biology and Biotechnology, University of Sheffield, Sheffield S10 2TN, UK, ^2^Department of Chemistry, Washington University, St. Louis, Missouri 63130-4889, USA, ^3^Department of Biochemistry and Molecular Biology, The Pennsylvania State University, University Park, PA 16802, USA

**Table of contents**

| **Table S1.** Sequences of PCR primers used for strain generation. | Page 3 |
| --- | --- |
| **Table S2.** Lifetime(s) of the (B)Chls in the B800 position in LH2 | Pages 4-5 |
| **Table S3.** (B)Chl lifetimes free in solvents | Page 6 |
| **Figure S1.** Absorption spectra and fits for (B)Chl *a* binding to WT-B850 only LH2 | Page 7 |
| **Figure S2.** Absorption spectra and fits for BChl *a* binding to (B)Chl reconstituted LH2s | Page 8 |
| **Figure S3**. Comparative Vis/NIR absorption spectra of WT and βR30L complexes containing (B)Chls in the B800 site following incubation in various (B)Chls | Page 9 |
| **Figure S4.** Spectra and fits for BChl *b* binding to (B)Chl reconstituted LH2s | Page 10 |
| **Figure S5.** Spectra and fits for Chl *d* binding to (B)Chl reconstituted LH2s | Page 11 |
| **Figure S6.** Spectra and fits for 3-acetyl Chl *a* binding to (B)Chl reconstituted LH2s | Page 12 |
| **Figure S7.** Spectra and fits for Chl *a* binding to (B)Chl reconstituted LH2s | Page 13 |
| **Figure S8.** Spectra of as-prepared WT, βR30L and βR30F LH2 complexes before and after incubation in excess BChl *a* | Page 14 |
| **Figure S9.** Spectra of WT, βR30L and βR30F B850 complexes following incubation in LDS at pH 8 | Page 14 |
| **Figure S10.** NIR-TA spectra and DADS for native βR30L and βR30F excited at 665 nm | Page 15 |
| **Figure S11.** NIR-TA spectra and DADS for native WT-LH2 excited at 800 nm, βR30L excited at 786 nm and βR30L LH2 excited at 783 nm | Page 16 |
| **Figure S12.** NIR TA spectra and DADS for native LH2 complexes excited at the same wavelengths as in Fig. 7 | Page 17 |
| **Figure S13.** Absorptance vs. excitation spectra for select (B)Chl-containing complexes | Page 18 |
| **Figure S14.** Chl emission spectra for reconstituted complexes native in buffer and solubilised in methanol | Page 19 |
| Supporting references | Page 20 |

**Table S1.** Sequences of PCR primers used for strain generation.

| Name | Sequence | Use |
| --- | --- | --- |
| BALMX KO Upstream F | CCGGAATTCGAACAACTTCTCGCTCGTCCAC | Knockout of *pufBALMX* |
| BALMX KO Upstream R | CTTGTCTCAGACAGCCATGCTATCCTCCGGATC |  |
| BALMX KO Downstream F | GATAGCATGGCTGTCTGAGACAAGTCTCGGGGC |  |
| BALMX KO Downstream R | CCCCAAGCTTGCAGGGTGCGCATCTGCTCG |  |
|  |  |  |
| *pufBALMX* screen F | GCCCTGGACCGCATCGTAGAGG | PCR screening of *pufBALMX* |
| *pufBALMX* screen R | CGCGTGGTTCAGTGCCTCGTAG |  |
|  |  |  |
| Puc1BA KO Upstream F | CCGGAATTCGCCAAGCCATCCTGAAATCTCG | Generation of βR30L LH2 |
| Puc1B R30L F | CTCGGCACCCTCGTCTTCGG |  |
| Puc1B R30L R | CCGAAGACGAGGGTGCCGAG |  |
| Puc1BA KO Downstream R | CCCCAAGCTTGTGTCGGACTTGAACCCGATCAG |  |
|  |  |  |
| R30F QuikChange F | CTCATCCTCGGCACCTTCGTCTTCGGTGGC | QuikChange PCR |
| R30F QuikChange R | GCCACCGAAGACGAAGGTGCCGAGGATGAG |  |
|  |  |  |
| Puc1BA screen F | CACGGCCATGTGCTGAAGATC | PCR screening of *puc1BA* |
| Puc1BA screen R | CACCGTCTGGATCGTGTGCAC |  |
|  |  |  |
| Puc2BA KO Upstream F | GCGTCTAGAGGCACCTACGAGCTCACCTTC | Knockout of *puc2BA* |
| Puc2BA KO Upstream R | AGAGGCTTATTGGGTCACTTGTACTCTCCCGAATGTG |  |
| Puc2BA KO Downstream F | GTACAAGTGACCCAATAAGCCTCTGCATTTCTCTGGGA |  |
| Puc2BA KO Downstream R | GTGCAAGCTTGTTCGCTACCTTGATCTTCGACATGG |  |
|  |  |  |
| Puc2BA Screen F | GGCCTCCTCAGACAGGAGGATG | PCR screening of *puc2BA* |
| Puc2BA Screen R | CACGCCATACTGCTTGATCGCATG |  |

**Table S2.** Lifetime(s) of the (B)Chls in the B800 position in LH2 used to obtain an amplitude weighted average (AWA). Single wavelength fits are at the position of stimulated emission for the (B)Chl.

| Complex and Fit Type | Fit λ  (nm) | τ_1_  (ps) | Fraction A_1_ | τ_2_  (ps) | Fraction  A_2_ | AWA τ  (ps) |
| --- | --- | --- | --- | --- | --- | --- |
| WT-Chl *a*-LH2 (665 nm exc) |  |  |  |  |  |  |
| Single wavelength | 674 | 4.3 | 0.73 | 12.2 | 0.27 | 6.5 |
| Single wavelength | 868 | 4.1 | 0.56 | 12.6 | 0.44 | 7.8 |
| Global (2 components) | 820-900 | 2.3 | 0.24 | 7.6 | 0.76 | 6.3 |
| Global (1 component) | 820-900 | 6.2 | 1.0 | - | - | 6.2 |
| *Avg* |  |  |  |  |  | *6.7+0.7* |
| R30F-Chl *a*-LH2 (665 nm exc) |  |  |  |  |  |  |
| Single wavelength | 674 | 3.7 | 0.61 | 12.2 | 0.39 | 7.0 |
| Single wavelength | 868 | 4.1 | 0.59 | 12.7 | 0.41 | 7.6 |
| Global (2 components) | 820-900 | 3.1 | 0.38 | 8.6 | 0.62 | 6.5 |
| Global (1 component) | 820-900 | 6.4 | 1.0 | - | - | 6.4 |
| *Avg* |  |  |  |  |  | *6.9+0.5* |
| R30L-Chl a-LH2 (665 nm exc) |  |  |  |  |  |  |
| Single wavelength | 674 | 3.1 | 0.48 | 7.7 | 0.52 | 5.4 |
| Single wavelength | 868 | 3.3 | 0.53 | 9.4 | 0.47 | 6.2 |
| Global (2 components) | 820-900 | 2.4 | 0.29 | 6.6 | 0.71 | 5.4 |
| Global (1 component) | 820-900 | 5.4 | 1.0 | - | - | 5.4 |
| *Avg* |  |  |  |  |  | *5.6+0.4* |
| 3-acetyl Chl *a*-LH2 (690 nm exc) |  |  |  |  |  |  |
| Single wavelength | 700 | 3.1 | 0.84 | 16.5 | 0.16 | 5.3 |
| Single wavelength | 868 | 2.3 | 0.63 | 11.4 | 0.37 | 5.6 |
| Global (2 components) | 820-900 | 2.2 | 0.59 | 8.1 | 0.41 | 4.6 |
| Global (1 component) | 820-900 | 4.9 | 1.0 | - | - | 4.9 |
| *Avg* |  |  |  |  |  | *5.1+0.5* |
| Chl *d*-LH2 (700 nm exc) |  |  |  |  |  |  |
| Single wavelength | 716 | 2.2 | 0.94 | 31.6 | 0.06 | 3.9 |
| Single wavelength | 868 | 1.4 | 0.66 | 6.5 | 0.34 | 3.1 |
| Global (2 components) | 820-900 | 2.3 | 0.79 | 12.9 | 0.21 | 4.6 |
| Global (1 component) | 820-900 | 3.0 | 1.0 | - | - | 3.0 |
| *Avg* |  |  |  |  |  | *3.5+0.7* |
| Chl *f*-LH2 (700 nm exc) |  |  |  |  |  |  |
| Single wavelength | 716 | 3.7 | 1.0 | - | - | 3.7 |
| Single wavelength | 868 | 2.5 | 1.0 | - | - | 2.5 |
| Global (1 component) | 820-900 | 2.0 | 1.0 | - | - | 2.0 |
| *Avg* |  |  |  |  |  | *2.7+0.8* |
| BChl *a*-LH2 (WT) (800 nm exc) |  |  |  |  |  |  |
| Single wavelength | 806 | 0.7 | 1.0 | - | - | 0.7 |
| Single wavelength | 868 | 0.4 | 1.0 | - | - | 0.4 |
| Global (1 component) | 820-900 | 0.5 | 1.0 | - | - | 0.5 |
| *Avg* |  |  |  |  |  | *0.5+0.2* |
| BChl *a*-βR30L (786 nm exc) |  |  |  |  |  |  |
| Single wavelength | 790 | 0.7 | 1.0 | - | - | 0.7 |
| Single wavelength | 868 | 0.5 | 1.0 | - | - | 0.5 |
| Global (1 component) | 820-900 | 0.5 | 1.0 | - | - | 0.5 |
| *Avg* |  |  |  |  |  | *0.6+0.1* |
| BChl *a*-βR30F (783 nm exc) |  |  |  |  |  |  |
| Single wavelength | 787 | 0.6 | 1.0 |  |  | 0.6 |
| Single wavelength | 868 | 0.4 | 1.0 | - | - | 0.4 |
| Global (1 component) | 820-900 | 1.5 | 1.0 | - | - | 1.5 |
| *Avg* |  |  |  |  |  | *0.8+0.6* |
| BChl *b*-LH2*^a^* (820 nm exc) | - | - | - | - | - | - |

*^a^*Limited by the response of the instrument.

**Table S3.** (B)Chl lifetimes free in solvents.

| Pigment | τ (ns) in pyridine | τ (ns) in toluene | τ (ns) average |
| --- | --- | --- | --- |
| Chl *a* | 6.3*^a^* | 6.2*^c^* | 6.3 |
| Chl *b* | 3.2*^a^* | 3.4*^c^* | 3.3 |
| Chl *d* | 6.2*^a^* | 6.7*^c^* | 6.5 |
| 3-acetyl-Chl *a* | 5.9*^b^* | 5.2*^b^* | 5.6 |
| BChl *a* | 2.9*^a^* | 3.1*^d^* | 3.0 |
| BChl *b* | 2.4*^a^* | - | 2.4 |
| Chl *f* | 5.6^e^ | - | 5.6 |

*^a^*From (1). *^b^*From this work. *^c^*From (2). *^d^*From (3). *^e^*From (4).

**Figure S1:** Vis/NIR absorption spectra (left) and plots of the A[Chl]/A850 ratio (see panel label) for (B)Chl binding to WT-B850 only LH2. A-B: BChl *a*, C-D: Chl *a*, E-F 3-acetyl-Chl *a*, G-H: Chl *d*, and BChl *b* I-J.

**Figure S2.** Vis/NIR absorption spectra (left) and plots of the A[Chl]/A850 ratio (see panel label) for BChl *a* binding to LH2 containing BChl *b* (A and B), Chl *d* (C and D), 3-acetyl-Chl *a* (E and F) and Chl *a* (G and H) within the B800 site.

**Figure S3**. UV/Vis/NIR absorption spectra of native WT LH2 (containing BChl *a* in the B800 site) (black) and LH2s reconstituted with BChl *b* (purple), Chl *d* (red), 3-acetyl-Chl *a* (blue) and Chl *a* (green) before (dashed lines) and after (solid lines) incubation in excess Bchl *b* (panel A), Chl *d* (panel B), 3-acetyl Chl *a* (panel C) and Chl *a* (panel D).

**Figure S4**. Vis/NIR absorption spectra (left) and plots of the A[Chl]/A850 ratio (see panel label) for BChl *b* binding to WT LH2 containing BChl *a* (A and B), Chl *d* (C and D), 3-acetyl-Chl *a* (E and F) and Chl *a* (G and H) in the B800 site.

**Figure S5.** Vis/NIR absorption spectra (left) and plots of the A[Chl]/A850 ratio (see panel label) for Chl *d* binding to WT LH2 containing BChl *a* (A and B), BChl *b* (C and D), 3-acetyl-Chl *a* (E and F) and Chl *a* (G and H) within the B800 site.

**Figure S6.** Vis/NIR absorption spectra (left) and plots of the A[Chl]/A850 ratio (see panel label) for 3-acetyl-Chl *a* binding to WT LH2 containing BChl *a* (A and B), BChl *b* (C and D), Chl *d* (E and F) and Chl *a*-LH2 (G and H) within the B800 site.

**Figure S7.** Vis/NIR absorption spectra (left) and plots of the A[Chl]/A850 ratio (see panel label) for Chl *a* binding to WT LH2 containing BChl *a* (A and B), BChl *b* (C and D), Chl *d* (E and F) and 3-acetyl-Chl *a* (G and H) within the B800 site.

**Figure S8:**Vis/NIR absorption spectra of WT (purple), βR30L (blue) and βR30F (red) LH2 complexes either as prepared (dashed lines) or following incubation in five-fold excess of Bchl *a* (solid lines).

**Figure S9.** Vis/NIR absorption spectra of WT (purple), βR30L (Blue) and βR30F (red) following LDS incubation and ion-exchange at pH 8.0. Note the retention of the B800 band for WT complexes, which is lost when pH is reduced to 5 (see Fig 3A, solid purple line).

**Figure S10.** NIR-TA spectra for native βR30L (Panels A and B) and βR30F (Panel D and E) excited at 665 nm with the rise of B850* bleaching in left-hand panels and decay in centre panels. The corresponding DADS are shown in right-hand panels (C and F).

**Figure S11.** NIR-TA spectra and DADS for BChl *a* B800 WT-LH2 excited at 800 nm (A-C), βR30L LH2 excited at 786 nm (D-F) and βR30F excited at 783 nm (G-I) with the rise of B850* bleaching in Panel A, D and G and decay in Panel B, E and H. The corresponding DADS are shown in panel C, F and I with DADS in red reflecting decay of B800* (primarily by energy transfer to B850) and DADS in blue reflecting decay of B800*.

**Figure S12.** NIR-TA spectra during growth or B850* (left panels) and decay of B850* (centre panels), and DADS (right panels) for Native WT LH2 excited at 690 nm (Panels A-C), 700 nm (Panels D-F), 705 nm (Panels G-I) and 820 nm (Panels J-L). Spectra are plotted on the same scales as those in Fig. 7 to allow direct comparison with the (B)Chl reconstituted samples.

**Figure S13.** Absorptance (red lines) vs. fluorescence excitation (blue lines) spectra for select (B)Chl-containing complexes and related controls. Spectra were normalized at B850.

**Figure. S14:** Chl *a* fluorescence emission spectra for reconstituted WT (A), βR30F (B) and βR30L (C) LH2 containing Chl *a* in the 800 site in buffer (purple) or in methanol (green). Panels E and D show spectra for wild-type containing Chl *d* and 3-acetyl-Chl *a*, respectively.

**Supplementary references:**

1. Niedzwiedzki, D. M., and Blankenship, R. E. (2010) Singlet and triplet excited state properties of natural chlorophylls and bacteriochlorophylls. *Photosynth. Res.* **106**, 227–238

2. Springer, J. W., Faries, K. M., Diers, J. R., Chinnasamy, M., Olga, M., Ling, K. H., Christine, K., Lindsey, J. S., Bocian, D. F., and Holten, D. (2012) Effects of substituents on synthetic analogs of chlorophylls. Part 3: The distinctive impact of auxochromes at the 7- versus 3-positions. *Photochem. Photobiol.* **88**, 651–674

3. Chen, C.-Y., Sun, E., Fan, D., Taniguchi, M., McDowell, B. E., Yang, E., Diers, J. R., Bocian, D. F., Holten, D., and Lindsey, J. S. (2012) Synthesis and physicochemical properties of metallobacteriochlorins. *Inorg. Chem.* **51**, 9443–9464

4. Niedzwiedzki, D. M., Liu, H., Chen, M., and Blankenship, R. E. (2014) Excited state properties of chlorophyll *f* in organic solvents at ambient and cryogenic temperatures. *Photosynth. Res.* **121**, 25–34
